# Unmet healthcare needs and the local extinction index: an analysis of regional disparities impacting South Korea’s older adults

**DOI:** 10.3389/fpubh.2024.1423108

**Published:** 2024-08-01

**Authors:** Younggyu Kwon, Minsung Sohn, Mankyu Choi

**Affiliations:** ^1^School of Health Policy and Management, College of Public Health Science, Korea University, Seoul, Republic of Korea; ^2^Center for Medical Education, College of Medicine, Chung-Ang University, Seoul, Republic of Korea; ^3^Division of Health and Medical Sciences, The Cyber University of Korea, Seoul, Republic of Korea; ^4^BK21 FOUR R&E Center for Learning Health Systems, Korea University, Seoul, Republic of Korea

**Keywords:** spatial analysis, spatial autocorrelation, hierarchical linear model, local extinction index, healthcare disparities, South Korea

## Abstract

**Background:**

This study examines the factors affecting unmet healthcare experiences by integrating individual-and community-level extinction indices.

**Methods:**

Using spatial autocorrelation and multilevel modeling, the study utilizes data from the Community Health Survey and Statistics Korea for 218 local government regions from 2018 to 2019.

**Results:**

The analysis identifies significant clustering, particularly in non-metropolitan regions with a higher local extinction index. At the individual level, some factors affect unmet medical needs, and unmet healthcare needs increase as the local extinction index at the community level increases.

**Conclusion:**

The findings underscore the need for strategic efforts to enhance regional healthcare accessibility, particularly for vulnerable populations and local infrastructure development.

## 1 Introduction

Despite extensive efforts to combat the declining birth rate in South Korea since 2006, the country still grapples with persistently low fertility rates and a rapid rise in its older adult population, creating a demographic challenge that threatens its socioeconomic stability. This issue varies between regions, with smaller cities and rural areas facing an escalating risk of depopulation (1, 2). Citing Masuda Hiroya’s warning about regional depopulation in Japan and Lee’s (3) study on local extinction in Korea, the 2019 Index of Local Extinction Risk by the Korea Employment Information Service indicates that over 100 municipalities in South Korea could face a risk of extinction by 2021, up from 97 in 2020. The Board of Audit and Inspection of Korea (4) predicts that by 2047, all 226 cities and counties could be at risk of extinction. Of these, approximately 70% are classified as having a “high extinction risk.” Given the persistent low birth rates in South Korea, a somber prediction suggests a steady decline by 2047, leading to a super-aged society where community functions deteriorate and the population decreases. Ultimately, this will negatively affect residents’ well-being (5).

The local extinction index is defined as the ratio of women aged 20–39 years to the population aged 65 years and older. This index is used to measure the risk of population decline, with lower values indicating higher risk due to lower birth rates and an aging population. The theoretical framework of local extinction involves understanding the socio-economic and demographic factors behind regional population declines, highlighting that the issue extends beyond aging and low birth rates (4). Regions facing severe local extinction often experience vulnerabilities in essential societal and economic aspects, such as housing, education, employment, healthcare, and welfare (6). This resource imbalance directly affects residents’ well-being by hindering basic human needs (7). Unfortunately, rural areas and smaller cities in South Korea grapple with social issues triggered by population changes, leading to growing healthcare disparities between regions (8). Here, the predominantly privatized healthcare system poses difficulties in providing essential medical services to underserved areas owing to financial constraints or closures of healthcare facilities in sparsely populated regions (9). Consequently, the issue of local extinction is no longer confined to rural and underdeveloped regions. Rather, it represents an ongoing and realistic challenge for the country.

Universal Health Coverage (UHC), a central tenet of the United Nations’ Sustainable Development Goals since 2015, remains a vital global health priority, aiming to ensure equal health rights for all individuals (10). UHC focuses on making healthcare services accessible to reduce health inequalities by improving factors like facilities, personnel, finances, and information related to healthcare provision and usage (11). Geographical accessibility, in this context, refers to the actual distance people need to travel to access healthcare, which is influenced by variables such as the distribution of healthcare facilities, transportation networks, and geographical barriers. Addressing health disparities stemming from limited geographical access necessitates collaborative national efforts (12, 13). Despite initiatives like the “First Basic Plan for Public Health and Medical Services” that aim to address regional healthcare disparities, gaps in unmet healthcare experiences due to geographical access disparities persist (14, 15). As non-urban areas witness population shifts, a comprehensive regional approach is vital to tackling health disparities in local communities to deal with impending challenges, such as local extinction (16).

Unmet healthcare refers to a situation in which medical needs, whether perceived by individuals or deemed necessary by medical professionals, are not met in a timely way (17). The importance of unmet healthcare needs can be categorized into three aspects. First, the absence of timely access to healthcare services is a violation of the fundamental right to health. Second, from a practical standpoint, not receiving necessary medical services can lead to health deterioration and increased mortality rates due to delayed diagnosis or treatment. This can escalate healthcare utilization and costs (18, 19). Unmet healthcare needs elevate disease severity and complications and diminish satisfaction with care and overall quality of life (20, 21). Third, from an equity perspective, healthcare disparities disadvantage vulnerable groups in terms of health, limiting their opportunities for better health and well-being (22).

Previous research predominantly approached unmet healthcare needs from an individual perspective, overlooking structural factors like societal and economic contexts (23–25). This limits effective policy changes and interventions. Currently, recognizing structural determinants as pivotal in shaping health decisions is gaining traction (26). While population shifts contribute to local extinction and resultant health issues, prior studies mainly focused on spatial distribution changes, neglecting the interplay between regional healthcare infrastructure and community health levels (27–29). However, addressing interregional health disparities arising from local extinction requires a balanced regional approach (30).

Given the aforementioned factors, this study primarily aimed to calculate the Local Extinction Risk Index, which will enable the monitoring of spatiotemporal trends in areas at risk of local extinction in South Korea. In addition, this study comprehensively investigates the influence of the extinction index on individuals’ access to healthcare services in different geographic locations. It is anticipated that the outcomes of this study will establish a robust foundation for shaping effective public health policies and optimizing resource allocation strategies. By addressing inter-regional health disparities resulting from variations in the Local Extinction Risk Index, the study aspires to offer well-informed and enduring policy recommendations aligned with our overarching objectives. These findings are particularly relevant to Sustainable Development Goal 3 (SDG3), which aims to ensure healthy lives and promote well-being for all ages (31). By addressing healthcare accessibility in regions with high risk, this study contributes to achieving SDG3 targets, such as reducing health disparities and ensuring UHC.

## 2 Materials and methods

This study examines the factors affecting unmet healthcare needs by integrating individual and community-level extinction indices. Using spatial autocorrelation and multilevel modeling, the study uses data from the Community Health Survey and Statistics Korea for 218 local government regions from 2018 to 2019.

### 2.1 Study area

South Korea has 17 regional jurisdictions, including seven metropolitan cities (Seoul, Busan, Daegu, Incheon, Gwangju, Daejeon, and Ulsan) and eight provinces (Gyeonggi-do, Gangwon-do, Chungcheongbuk-do, Chungcheongnam-do, Jeollabuk-do, Jeollanam-do, Gyeongsangbuk-do, and Gyeongsangnam-do). The country also has a special self-governing province of Jeju and a self-governing city of Sejong, which cover the capital region. These entities are subdivided into smaller units like cities, counties, and districts. For the spatial analysis in this study, 218 of 226 local government entities were selected, excluding islands and remote areas, based on data availability.

### 2.2 Variables

Table 1 presents the variable definitions.

**Table 1:** Definitions of the variables.

Variables		Variable Meaning		Source
**1-Level (Individual level)**				
Y		1 = The percentage of those aged 65 years and above who answered “yes” to the question “During the past year, have you ever been unable to go to a hospital or clinic (not including a dentist) when you wanted to?” 0 = Other response		Community Health Survey (2018, 2019)
Predisposing factors	Sex	1 = Men 0 = Women		
	Age	Over 65 years		
	Education	5 = University 4 = High school 3 = Middle school2 = Elementary school 1 = None		
	Marital status	1 = Spouse 0 = Separation, Divorce, Break-up		
Enabling factors	Income	Household Income Equalized Monthly Income		
Need factors	Self-rated health	5 = Very good 4 = Good 3 = Fair2 = Poor 1 = Very poor		
	Chronic disease	1 = Chronic disease (Hypertension or Diabetes) 0 = None		
**2-Level (Community level)**				
Local Extinction Index	LEI	2- (Number of women aged 20–39 years / Population aged 65 years and above)		Korea Statistical Information Service (2018, 2019)

#### 2.2.1 Dependent variable

Unmet healthcare refers to situations where individuals, despite having a desire and need for medical care, experience a lack of access to healthcare services for any reason. This state is an “unmet healthcare experience” (32). The variable for unmet healthcare needs is defined based on the proportion of individuals aged 65 years and older who, in a community health survey, responded “yes” to the following question: “In the past year, have you needed medical treatment (examinations or therapy) at a clinic (excluding dental care) but not received it?”

#### 2.2.2 Independent variables

##### 2.2.2.1 Individual-level variables

The independent variables were categorized based on Andersen’s (33) healthcare utilization model, which includes predisposing factors, enabling factors, and need factors. In this study, predisposing factors included gender, age, educational level, and marital status. Educational levels were classified as “No education,” “Elementary school,” “Middle school,” “High school,” and “University.” Marital status was categorized as “Married spouse” and “Non-spouse.” Enabling factors included monthly household income adjusted for equivalence. Need factors included self-rated health status and chronic illness. Self-rated health status was categorized as “Very bad,” “Bad,” “Fair,” “Good,” and “Very good.” Finally, chronic illness (hypertension or diabetes) was classified as “Present” or “Absent.”

##### 2.2.2.2 Community-level variables

The local extinction index employed the number of women aged 20–39 years, based on reproductive capability, to gauge potential regional decline. According to a previous study, a 50% decrease in this number could render a locality “at risk” because of the resultant challenges in social security and employment (34). Lee (3) extended Masuda’s method, assessing the decline risk through the ratio of women aged 20–39 years to those aged 65 years and older. An index ≥1.0 signifies an area is “safe,” 0.6–1.0 suggests it is in “incipient decline,” 0.2–0.5 flags it as “at risk,” and < 0.2 implies an area is “high risk” (3). The formula for the decline index in this study is:

$$\mathrm{Local}\;\mathrm{Extinction}\;\mathrm{Index}=\frac{\mathrm{Women}\;\mathrm{Aged}\;20-39\;\mathrm{Years}}{\mathrm{Population}\;\mathrm{Aged}\;65\;\mathrm{Years}\;\mathrm{and}\;\mathrm{Above}}$$

The numerator of the local extinction index signifies women aged 20–39 years and reflects low birth rates. The denominator represents the older adult population (65 years and older), which will indicate population aging. Emphasizing low birth rates and the growing older adult population leads to lower index values, denoting a more pronounced population decline and a higher risk of regional decline. For interpretation, this study utilized a fixed value, in which higher local extinction index values indicate higher risk.

In this study’s framework, a higher local extinction index indicates higher risk and is linked to greater unmet healthcare needs due to demographic imbalances like low birth rates and an aging population. The extinction index serves as a crucial variable for predicting and addressing healthcare needs in aging populations, helping policymakers allocate resources effectively and develop strategies to mitigate the impacts of demographic changes on healthcare systems.

### 2.3 Analysis methods

Statistical analyses of the existing data were performed using RStudio (Version 4.2.3, Windows) and GeoDa (Version 1.20.0.10). To visualize spatial patterns, QGIS (Version 3.24.1) was used.

#### 2.3.1 Spatial autocorrelation

Spatial data refers to the interdependence and interaction between geographical spaces with similar characteristics, which tend to be highly correlated as they are spatially adjacent (35). We used Moran’s *I* statistic (36) to analyze the univariate spatial autocorrelation. The basic formula is given by Eq. (1):

(1) $$\mathrm{Mora}n^{\prime }s\;I=\frac{N\sum_{i=1}^{n}\sum_{j=1}^{n}\omega_{ij}\left(Y_{i}-\bar{Y}\right)\left(Y_{j}-\bar{Y}\right)}{\left(\sum_{i=1}^{n}\sum_{j=1}^{n}\omega_{ij}\right)\sum_{i=1}^{n}{\left(Y_{i}-\bar{Y}\right)}^{2}}$$

where N represents the number of spatial units, Y is the dependent variable, Y_i_ denotes the mean value of Y in unit i, and ω represents the spatial weight matrix at locations i and j. Moran’s I index is used to assess statistical significance through a Z-test. Its basic formula is given as Eq. (2):

(2) $$Z=\frac{I-E\left(I\right)}{S_{e}\left(I\right)}$$

where E(I) and S_e_ (I) represent the mean and standard deviation of statistic I, respectively. The spatial autocorrelation index of Moran’s I ranges from −1 to +1, with values closer to +1 and − 1 indicating a more positive and negative spatial correlation, respectively (37).

We used local indicators of spatial associations to calculate hotspots in each city, revealing four main patterns: high-high (HH), high-low (HL), low-high (LH), and low-low (LL). HH means the variable is elevated both within the area and nearby. HL indicates high values within one area but low values in the surroundings. LH suggests lower values in one area compared to its surroundings, while LL implies low values in both the area and its surroundings.

#### 2.3.2 Hierarchical linear model

This study employed a hierarchical linear model (HLM) to analyze the impact of the local extinction index variables and individual-level socio-demographic factors on the unmet healthcare needs of older adults. HLM, unlike conventional regression models, considers individual and higher-level contextual factors simultaneously, yielding more accurate outcomes (38). It acknowledges the interplay between these levels, thus enhancing the reliability of results (38). To determine the suitability of HLM, an intraclass correlation coefficient (ICC) exceeding 5% was recommended in the presence of group-level differences (39). Eq. (3) presents the formula:

(3) $$ICC=\frac{\tau }{\tau +\sigma^{2}}$$

where τ represents the variance at the regional level and σ^2^ the variance at the individual level. ICC represents the proportion of total variance in the dependent variable attributed to between-region differences, indicating regional disparities (40). Therefore, ICC allows us to understand the proportion of variance in the overall data explained by regional disparities.

In the course of our study, the HLM analysis approach was adopted. This involved the implementation of both individual-level and regional-level models. The individual-level model was designed to delve into the intricacies of unmet healthcare needs. This model meticulously dissected the unique attributes of each individual, effectively capturing the nuanced fluctuations in healthcare requirements across various geographical domains. Concomitantly, the regional-level model was crafted to provide a broader perspective. This model systematically probed the underlying determinants contributing to unmet healthcare needs within distinct geographic areas. It thoughtfully factored in regional idiosyncrasies and influences that collectively shape healthcare needs within specific locales.

The individual-level and regional-level models are given by Eqs. (4) and (5):

(4) $$Y_{ij}=\beta_{0j}+\gamma_{ij}$$

(5) $$\beta_{0j}=\gamma_{00}+\mu_{0j}$$

**Figure 1:**
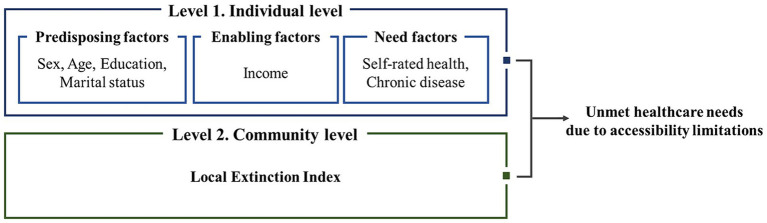
Research model.

The individual-level model (Eq. 4) represents the unmet healthcare needs ( $Y_{ij}$ ) of older adults. It combines the average needs in their region ( $\beta_{0j}$ ) with an individual-specific error ( $\gamma_{ij}$ ). The regional-level model (Eq. 5) examines how the characteristics of an individual’s region affect the outcome. $\beta_{0j}$ is the average unmet healthcare need in region j. Specifically, $\gamma_{00}$ is a constant representing the overall average across all regions, and $\mu_{0j}$ is a region-specific error reflecting unique regional traits that contribute to individual variability beyond the average. Here, the distinct cultural, economic, and educational factors in each region can influence the outcome. The HLM uses individual and regional variables to predict outcomes, offering a valuable approach to analyzing regional disparities and individual differences. Figure 1 illustrates the model of this study.

### 2.4 Ethics statement

The approval for this study was obtained by the Institutional Review Board of Korea University (approval number: KUIRB-2023-0174-01), in compliance with the Helsinki Declaration.

## 3 Results

**Figure 2:**
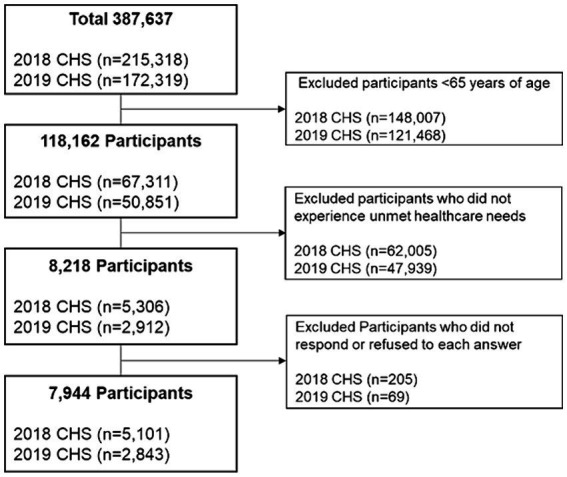
Data selection flow chart.

In this study, individual-level analysis was conducted using community health survey data from 2018 and 2019. The survey included 67,311 individuals aged 65 years and above in 2018 and 50,851 in 2019. Following the exclusion of participants who did not report medical dissatisfaction, the final dataset for analysis comprised 5,101 participants from 2018 and 2,843 from 2019, as illustrated in Figure 2.

### 3.1 Ten regions with the highest unmet healthcare needs

Table 2 displays the five leading and trailing areas according to the local extinction index, categorized by city and county. The top five regions in 2018 and 2019 were certain parts of Gyeongsangbuk-do, Gyeongsangnam-do, and Jeollanam-do. The bottom five regions in 2018 included Ulsan, Daejeon, specific zones in Gyeonggi-do, and certain areas of Gyeongsangbuk-do. In 2019, the bottom five included Ulsan, Gyeonggi-do, Daejeon, and certain parts of Gyeongsangbuk-do.

**Table 2:** Regions at the Top and Bottom of the Local Extinction Index.

Years	2018		2019	
No.	“Top 5” Regions	LEI	“Bottom 5” Regions	LEI
1	Goheunggun, Jeollanam-do	1.89	Goheunggun, Jeollanam-do	1.9
2	Uiseonggun, Gyeongsangbuk-do	1.89	Uiseonggun, Gyeongsangbuk-do	1.9
3	Gunwigun, Gyeongsangbuk-do	1.88	Gunwigun, Gyeongsangbuk-do	1.89
4	Hapcheongun, Gyeongsangnam-do	1.88	Hapcheongun, Gyeongsangnam-do	1.89
5	Cheongsonggun, Gyeongsangbuk-do	1.87	Boseonggun, Jeollanam-do	1.88

No.	“Bottom 5” Regions	LEI	“Bottom 5” Regions	LEI
1	Bukgu, Ulsan	0.48	Bukgu, Ulsan	0.55
2	Osansi, Gyeonggi-do	0.57	Osansi, Gyeonggi-do	0.67
3	Yuseonggu, Daejeon	0.6	Hwaseongsi, Gyeonggi-do	0.69
4	Gumisi, Gyeongsangbuk-do	0.65	Yuseonggu, Daejeon	0.71
5	Hwaseongsi, Gyeonggi-do	0.66	Gumisi, Gyeongsangbuk-do	0.77

The choropleth map of the local extinction index offers insights into spatial distribution, with a primary focus on non-capital regions (Appendix A). All areas experienced an increased risk of regional decline from 2018 to 2019. Jeollanam-do exhibited the highest risk of decline, and Sejong City had the lowest risk (Appendix B).

### 3.2 Descriptive statistics

Table 3 presents the descriptive statistics of the variables. Regarding the dependent variable, unmet healthcare needs owing to accessibility limitations decreased slightly, from 27% in 2018 to 23% in 2019. The mean age increased slightly, from 75.84 years in 2018 to 76.01 years in 2019, and household income also increased from 4.95 to 5.09, respectively. The ratio of women was higher than that of men. Women also had a higher prevalence of chronic diseases. The proportion of married individuals was higher in 2018 at 50.6% than that of unmarried individuals. However, in 2019, the proportion of unmarried individuals was higher at 55.7%. Education level was highest for “elementary school,” followed by “no education,” “middle school,” “high school,” and “college” in descending order. Regarding participants’ self-rated health status, a large proportion rated it as “poor,” followed by “fair,” “very poor,” “good,” and “very good” in that order.

**Table 3:** Descriptive Statistics.

Years		2018 (*N* = 5,101)			2019 (*N* = 2,843)		
Variables		Mean(SD)	Min	Max	Mean(SD)	Min	Max
**Individual level (Level 1)**							
Predisposing factors	Age	75.84 (6.97)	65	106	76.01 (7.12)	65	99
Enabling factors	Income	4.95 (2.29)	0	11.4	5.09 (2.33)	2.4	11.4
**Community level (Level 2)**							
Local Extinction Index		1.56 (0.3)	0.48	1.89	1.6 (0.29)	0.55	1.9

Years			2018 (*N* = 5,101)	2019 (*N* = 2,843)
Variables			Frequency (%)	Frequency (%)
**Individual level (Level 1)**				
Unmet healthcare needs due to accessibility: yes			1,379 (27)	664 (23)
Unmet healthcare needs due to accessibility: no			3,722 (73)	2,179 (77)
Predisposing factors	Gender	Men	1,481 (29)	424 (15)
		Women	3,620 (71)	2,419 (85)
	Education	University	150 (3)	62 (2)
		High school	443 (9)	210 (7)
		Middle school	623 (12)	326 (11)
		Elementary school	2,319 (45)	1,283 (45)
		No education	1,566 (31)	962 (35)
	Marital status	Spoused	2,582 (51)	1,259 (44)
		Separation, Divorce, Breakup	2,519 (49)	1,584 (56)
Need factors	Self-rated health	Very good	37 (0.7)	16 (0.6)
		Good	519 (10)	226 (8)
		Fair	1,402 (27)	730 (25)
		Poor	2,006 (39.3)	1,180 (42)
		Very poor	1,137 (23)	691 (24.4)
	Chronic disease	Chronic disease: yes	3,196 (63)	1,794 (63)
		Chronic disease: no	1,905 (37)	1,049 (37)

### 3.3 Spatial autocorrelation of the local extinction index

**Figure 3:**
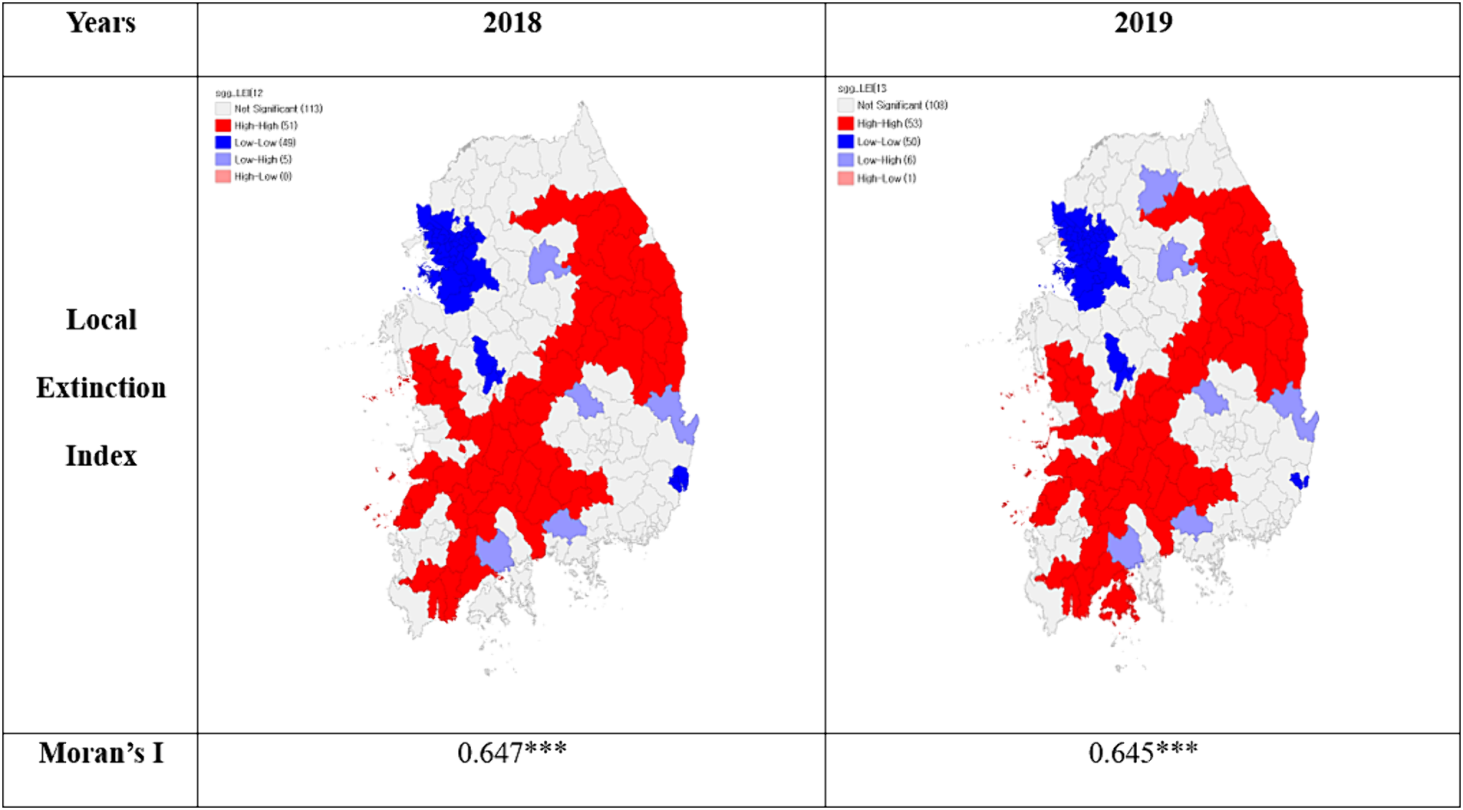
Spatial autocorrelation of the local extinction index. *p < 0.05, **p < 0.01, ***p < 0.001.

To calculate Moran’s *I* coefficient, a spatial weighting matrix was constructed to ensure that each location had at least one neighboring point. Figure 3 shows the results of the spatial autocorrelation. The computed and verified Moran’s I values demonstrated statistical significance for both years, yielding values of 0.647 and 0.645 in 2018 and 2019, respectively.

For spatial clustering, of the 218 administrative districts across the country, the number of HH clusters increased from 51 regions in 2018 to 53 in 2019. HH clusters were observed in specific areas of Gangwon-do, Gyeongsangbuk-do, Gyeongsangnam-do, Chungcheongbuk-do, Chungcheongnam-do, Jeollabuk-do, and Jeollanam-do. The number of LL clusters increased from 49 regions in 2018 to 50 in 2019, with LL clusters identified in certain parts of Seoul, Gyeonggi-do, Incheon, Daejeon, Sejong, and Ulsan.

### 3.4 Results of the hierarchical linear model

Table 4 displays the results from the HLM’s null model analysis. An ICC, which shows regional-level variance within the total variance, was evaluated to test the validity of the model. The ICC values were around 11% in 2018 and 8.9% in 2019. Thus, validity was confirmed as the ICC values exceeded the 5% threshold (39). A comparison of the variance components showed an increase in total individual variance from 1.8 to 2.3%, likely owing to differing local extinction index.

**Table 4:** Results of the null model.

Years	2018		2019	
Fixed effects	Coff.	S.E.	Coff.	S.E.
$\gamma_{{\;}_{00}}$	−1.377***	0.321	−1.451***	0.294
Random effects				
$\mu_{{\;}_{0j}}$	0.407		0.3218	
$r_{{\;}_{ij}}$	0.638		0.5673	
$x^{2}$	364.14***		275.15***	
ICC	11%		8.9%	

*p < 0.05, **p < 0.01, ***p < 0.001; ICC, Intraclass correlation coefficient.

Table 5 provides the results of the multilevel model analysis. Significant influences (*p* < 0.001) on older adults’ unmet healthcare experiences by geographical area included gender, age, education, income, self-rated health, and marital status in 2018, and age, education, marital status, self-rated health, and chronic illnesses in 2019. The local extinction index consistently demonstrated a significant impact (*p* < 0.001) on older adults’ healthcare experiences.

**Table 5:** Results of the hierarchical linear model.

Years	2018 (*N* = 5,101)				2019 (*N* = 2,843)			
Fixed effects	Model 1		Model 2		Model 1		Model 2	
Var.	Beta	SE	Beta	SE	Beta	SE	Beta	SE
**Individual level (Level 1)**								
Intercept	−1.49***	0.35	−1.51***	0.35	−1.31***	0.33	−1.66***	0.33
Gender	−0.36***	0.09	−0.36***	0.09	−0.22	0.16	−0.22	0.16
Age	0.07***	0.01	0.07***	0.01	0.06***	0.01	0.06***	0.01
Education	−0.14***	0.04	−0.13***	0.04	−0.14**	0.06	−0.12*	0.06
Marital status	0.01	0.08	0.02	0.08	−0.31***	0.11	−0.31***	0.11
Income	−0.03**	0.02	−0.04**	0.02	−0.01	0.02	−0.12	0.02
Self-rated health	−0.11***	0.04	−0.11***	0.04	−0.36***	0.06	−0.35***	0.06
Chronic disease	0.1	0.07	0.1	0.07	0.33***	0.1	0.34***	0.1
**Community level (Level 2)**								
Local Extinction Index			1.85***	0.39			2.33***	0.62

Random effects	Var	SD	Var	SD	Var	SD	Var	SD
Regional Index	0.4784	0.6916	0.4874	0.6981	0.3489	0.5906	0.4154	0.6445
AIC	5406.2		5385.4		2741.4		2729.5	
BIC	5,465		5450.7		2794.4		2789.0	

*p < 0.05, **p < 0.01, ***p < 0.001, AIC, Akaike Information Criterion; BIC, Bayesian Information Criterion.

### 3.5 Model fit evaluation

This study aimed to assess the final model’s goodness of fit by comparing two models: Model 1, which considered individual-level factors, and Model 2, which included regional-level factors. Model fit was evaluated using Akaike’s Information Criterion [AIC; see (41)] and Bayesian Information Criterion (BIC) indices, where lower values indicate a better-fitting and less complex model. In general, a difference in the AIC value of more than 4 suggests that the model needs improvement (42, 43). For 2018, Model 1 had an AIC/BIC of 5406.2/5465, and Model 2 had an AIC/BIC of 5385.4/5450.7. In 2019, Model 1 had an AIC/BIC of 2741.4/2794.4, and Model 2 had an AIC/BIC of 2729.5/2789. As such, the comparative analysis showed Model 2 to be a more suitable fit for the data.

## 4 Discussion

Recent research on regional health disparities extends beyond identifying gaps to understanding their impact on residents’ well-being. Prior studies commonly visualized changes in depopulating regions without fully explaining local health disparities. Furthermore, research on unmet healthcare needs typically focused on individuals and overlooked structural influences such as social and economic contexts, resulting in a limited understanding. This study is novel in that it integrates Andersen’s healthcare utilization model and incorporates individual and regional factors to comprehensively grasp forces affecting unmet healthcare experiences. This study employed spatial analysis and multilevel modeling with nationwide data (2018–2019) from the Community Health Survey and Statistics Korea.

Analyzing the spatial autocorrelation of the local extinction index, an increase in clustering was observed, especially in non-metropolitan areas, from 51 regions in 2018 to 53 in 2019. This aligns with earlier research indicating that areas facing local extinction are predominantly distributed in non-metropolitan regions such as Jeollabuk-do, Jeollanam-do, Gyeongsangbuk-do, and Gyeongsangnam-do (26). The spatial clustering emphasizes the need for targeted policy interventions to address the unique challenges in these areas.

The results from the multilevel model show the following. First, individual characteristics revealed important insights into geographical disparities in unmet healthcare needs. Gender emerged as a determinant, with women being more vulnerable, which is consistent with prior research (13, 44, 45). Age also had a significant role, in that that older individuals faced higher odds of such experiences. This is consistent with the findings of Jang and Lee (46) and Kwon and Choi (47), who both emphasized rational healthcare resource distribution for aging-related challenges. In addition, education had an impact: a higher level of education was linked to increased odds of unmet healthcare needs, which is also supported by previous studies (13, 48–50). In 2019, the presence of a spouse was an influencing factor, underscoring the importance of paying attention to healthcare for individuals without a spouse (45). These findings stress the need for ensuring gender-specific interventions, rational resource allocation, and addressing educational disparities to improve healthcare accessibility and management.

Second, concerning enabling factors, higher household income was linked to reduced geographical disparities in unmet healthcare needs in 2018. This is similar to previous research highlighting the elevated likelihood of such issues among lower-income households owing to limited accessibility (51–53). Here, income inequality exacerbates healthcare disparities, leading to more unmet healthcare needs among lower-income groups (51). Similarly, Kim et al. (52) found a higher prevalence of unmet healthcare needs in lower-income households. Song et al. (51) also reported an odds ratio of 4.95 (95% CI 1.91–12.87) for unmet healthcare needs in lower income groups compared to higher income groups. These findings underscore the persistent role of economic factors in shaping unmet healthcare needs. While statistical significance was observed only for 2018, future research could delve into income categorization for a more nuanced understanding. Nonetheless, acknowledging the impact of economic factors, it is essential to explore interventions such as strengthening health insurance coverage and improving payment systems to alleviate financial burdens.

Third, regarding need factors, better self-perceived health was linked to reduced geographical disparities in unmet healthcare needs, consistent with Choi and Ryu (45) and Kim et al. (52). Choi and Ryu (45) associated better health with lower odds (0.34; 95% CI, 0.26–0.44) of experiencing unmet healthcare needs. Kim et al. (52) also found that self-perceived health influenced unmet healthcare needs. They confirmed that poorer self-perceived health may lead to lower health behavior adherence, causing individuals to perceive themselves as unhealthy if their healthcare needs are not met, in contrast with those who perceive better health (54). The presence of chronic illnesses increased the odds of geographical disparities in unmet healthcare needs, which is consistent with the results of prior research (24, 55). While Kim and Lee (55) emphasized the impact of chronic illnesses, Kim and Eun (24) did not find a significant effect in panel data from 2009 to 2013. This study found the data for 2019 to be significant, and given Korea’s aging population, addressing geographical health disparities necessitates policies targeting vulnerable chronic illness groups.

Fourth, disparities in the local extinction index significantly contributed to higher individual-level geographical unmet healthcare experiences. The spatial autocorrelation analysis revealed that areas with higher local extinction indices, primarily rural regions, experienced limited healthcare access, which is consistent with prior research (8, 56, 57). This highlights the importance of addressing structural healthcare inequalities in rural and high-risk regions to ensure equitable access to healthcare services. An et al. (8) emphasized rural–urban healthcare resource disparities impacting accessibility due to transportation challenges. Similarly, Chang (56) highlighted the influence of poor healthcare service accessibility on unmet healthcare needs among older adults. Taqi et al. (57) underscored the role of rural healthcare infrastructure deficits in hindering access, revealing geographical disparities in unmet healthcare needs. Therefore, enhancing healthcare infrastructure and improving transportation networks in these regions is a crucial piece of addressing these unmet healthcare disparities. By enhancing healthcare accessibility in regions with high local extinction indices, this study supports SDG3 (reduce health disparities and ensure universal health coverage). The findings underscore the necessity for comprehensive policy interventions aligned with the WHO and UN guidelines to address regional healthcare disparities. Specifically, improving healthcare infrastructure and transportation in high-risk areas can significantly enhance health outcomes and promote a more equitable healthcare system. The WHO’s framework on social determinants of health emphasizes the importance of improving healthcare accessibility to reduce health inequities (26). In addition, UNDP’s report on sustainable development highlights the need for robust infrastructure and transportation networks in underserved regions to achieve SDG3 (58). These international guidelines align with the study’s recommendations, advocating for targeted policy interventions to ensure equitable healthcare access and improve overall health outcomes in high-risk areas.

In conclusion, this study revealed that individual-level factors, along with the community-level risk of local extinction, contribute to increased geographical disparities in unmet healthcare needs among older adults. Despite government efforts to enhance healthcare accessibility under the “Public Health and Medical Service Act,” the findings of this study highlight variations in perceived accessibility constraints based on the degree of local extinction. Specifically, considering the exacerbated geographical unmet healthcare disparities due to differences in the local extinction index, addressing economic accessibility and developing healthcare systems and infrastructure within local communities are essential to mitigating inter-regional healthcare inequalities. As an example of how to address these issues, Japan introduced the concept of a “community living district” in 2014 to combat population decline and local extinction. These districts integrate daily facilities for healthcare, welfare, and shopping to restructure regions, fostering connections between neighboring clusters and enhancing well-being (59, 60). Furthermore, politician and scholar Masuda Hiroya proposed “core cities” to encourage growth around metropolitan regions, advocating for continuous policy efforts (61). In response, Korea designated 89 high-risk municipalities as population decline regions and introduced the “Special Act on Population Decline Area Support” to prevent population disappearing, emphasizing comprehensive support (8). However, managing areas beyond this law is crucial owing to widespread population decline. To mitigate geographical disparities in unmet healthcare needs, a growth-centric “community living district” could link regional resources. Addressing the vulnerable older adult population as Korea becomes an ultra-aged society requires community-centered health systems to bridge health disparities between urban and rural areas. Furthermore, despite its contributions, this study also has limitations. First, using yearly samples from the Community Health Survey instead of panel data may hinder establishing robust causal relationships owing to changing sample composition. Future regional panel surveys could offer deeper insights into long-term trends and higher-level causal links. Second, the exclusive reliance on the local extinction index on population data for discussing local extinction might narrow its scope. Incorporating broader regional characteristics like economic activity and education level could provide a more accurate assessment. Third, the scope of this study was limited as it excluded islands and rural areas from the spatial analysis. Furthermore, data adjustments may affect the generalizability of the results. Geographical data often exhibit spatial dependence, as linked to the Modifiable Areal Unit Problem (MAUP) (62), potentially raising issues related to the selection of spatial units.

Nonetheless, this study boasts several noteworthy strengths. First, it combines individual-level variables with regional data, creating a diverse dataset that captures interactions between individuals and their local communities. This comprehensive approach provides insights into education, income, health, and the environment, enabling tailored policies for socioeconomic revitalization and healthcare promotion that align with regional traits. Second, incorporating the hierarchical structure of the local extinction index enhances the precision of the model. Unlike traditional linear regression, multilevel models consider interactions within this hierarchy, leading to more accurate results. The correlation between the local extinction index and geographical disparities in unmet healthcare needs is explored, offering informed policy recommendations for health policy formulation. Third, analyzing time-series data shows temporal changes and trends in regions. Over time, the influence of the local extinction index and individual-level factors may evolve, allowing predictions about geographical disparities in unmet healthcare issues stemming from local extinction. These insights are valuable for enhancing health policies, improving community well-being, and addressing healthcare disparities.

## 5 Conclusion

This study differs from previous research in that it comprehensively analyzes individual-level key factors based on Anderson’s healthcare utilization model and the community-level local extinction index to clarify the factors influencing unmet medical experiences. The results of this study are expected to contribute to improving health disparities in local communities if a system for community-centered, geographically-based healthcare management is established. Furthermore, it is expected that focusing on vulnerable areas and designating “cluster living zones” could reduce the gap in unmet medical needs across regions by ensuring that resources are allocated for social infrastructure development and enhancement. Addressing healthcare accessibility in high local extinction index regions is essential for achieving SDG3, which aims to reduce health disparities and ensure universal health coverage. Policy interventions aligned with WHO and UN guidelines can effectively mitigate regional healthcare disparities, significantly improving health outcomes and overall well-being.

Regional decline in South Korea is currently worsening a grave crisis. This predicament is expected to adversely affect the health and welfare of local community residents owing to future population decline, economic downturns, and worsened accessibility to medical services. Consequently, the government and relevant organizations should formulate strategies to enhance medical infrastructure and foster collaboration and community awareness within local communities.

## Data availability statement

The original contributions presented in the study are included in the article/Supplementary material, further inquiries can be directed to the corresponding author.

## Ethics statement

The studies involving humans were approved by the Institutional Review Board of Korea University (approval number: KUIRB-2023-0174-01). The studies were conducted in accordance with the local legislation and institutional requirements. Written informed consent for participation was not required from the participants or the participants’ legal guardians/next of kin in accordance with the national legislation and institutional requirements.

## Author contributions

YK: Data curation, Formal analysis, Investigation, Visualization, Writing – original draft, Conceptualization. MS: Conceptualization, Writing – review & editing. MC: Conceptualization, Supervision, Writing – review & editing.

## References

[1] Choi JH Park PG. Regional characteristics of the shrinking cities in Korea and its implication toward urban policies. J Korean Urban Geogr Soc. (2020) 23:1–13. doi: 10.21189/JKUGS.23.2.1

[2] Yim S. Local small cities’ population reduction and the characteristics of their growth and decline. J Korean Geogr Soc. (2019) 54:365–86.

[3] Lee SH (2016). Seven analyses on regional extinction in Korea. Regional Employment Trend Brief p. 4–17. Available at: https://www.keis.or.kr/user/extra/main/2405/publication/reportList/jsp/LayOutPage.do?categoryIdx=129&pubIdx=2237&reportIdx=3362&spage=3 (Accessed March 8, 2016).

[4] The Board of Audit and Inspection of Korea. Audit report (response to population structure changes I [regional]). Board of Audit and Inspection (2021) Available at: https://www.bai.go.kr/bai/result/branch/detail?srno=2622 (Accessed 13 August 2021).

[5] Ju SH. Local government depopulation status and policy alternatives. Korean Assoc Local Gov Admin Stud. (2021) 35:295–321.

[6] Lim SH Hong SJ (2019). Revitalizing demand-responding mobility service in declining local cities. The Korea Transport Institute. 1–195 https://www.koti.re.kr/user/bbs/BD_selectBbs.do?q_bbsCode=1017&q_bbscttSn=20200522134315011&q_clCode=1#section1 (Accessed November 30, 2019).

[7] Shin HS Lee SH Park BH (2012). Healthcare delivery reform for reducing health inequality. Korea Institute for Health and Social Affairs. Available at: https://repository.kihasa.re.kr/handle/201002/9763.

[8] An S Kim NH Kim YN (2019). Comparison of health status and the effectiveness of health cost between rural and urban residents. Korea Rural Economic Institute. https://www.krei.re.kr/krei/researchReportView.do?key=67&pageType=010101&biblioId=523289&pageUnit=10&searchCnd=all&searchKrwd=&pageIndex=7 (Accessed March 13, 2003).

[9] National Assembly Research Service. NARS issue of the year 2023 (2022). https://www.nars.go.kr/report/view.do?cmsCode=CM0073&brdSeq=41053 (Accessed December 30, 2022).

[10] Kang MA. Theoretical principles and practical approaches to universal health coverage. Health Insurance Rev Assessment Serv HIRA Policy Trends. (2016) 10:16–26.

[11] O’Connell T Rasanathan K Chopra M. What does universal health coverage mean? Lancet. (2014) 383:277–9. doi: 10.1016/S0140-6736(13)60955-1 23953765

[12] Cho HJ. Equity in health care: current situation in South Korea. J Korean Med Assoc. (2013) 56:184–94. doi: 10.5124/jkma.2013.56.3.184

[13] Garchitorena A Ihantamalala FA Révillion C Cordier LF Randriamihaja M Razafinjato B. Geographic barriers to achieving universal health coverage: evidence from rural Madagascar. Health Policy Plan. (2021) 36:1659–70. doi: 10.1093/heapol/czab087, PMID: 34331066 PMC8597972

[14] Kim JE Hahm MI. Association between residential area and unmet healthcare needs due to physical accessibility. Health Policy Manag. (2021) 31:197–206. doi: 10.4332/KJHPA.2021.31.2.197

[15] Yim J. Measures for strengthening public health care. Korea Institute Health Social Affairs. (2022). doi: 10.23062/2022.09.2

[16] Chang IS (2020). Regional demographic changes and policy responses in Korea. Korea Institute for Health and Social Affairs. Available at: https://repository.kihasa.re.kr/handle/201002/37180

[17] Aday LA Andersen R. A framework for the study of access to medical care. Health Serv Res. (1974) 9:208–20. PMID: 4436074 PMC1071804

[18] Alonso J Orfila F Ruigómez A Ferrer M Antó JM. Unmet health care needs and mortality among Spanish elderly. Am J Public Health. (1997) 87:365–70. doi: 10.2105/AJPH.87.3.365, PMID: 9096535 PMC1381006

[19] Tidikis F Strasen L. Patient-focused care units improve service and financial outcomes. Healthc Financ Manage. (1994) 48:38–40, 42, 44. PMID: 10146062

[20] Allin S Grignon M Le Grand J. Subjective unmet need and utilization of health care services in Canada: what are the equity implications? Soc Sci Med. (2010) 70:465–72. doi: 10.1016/j.socscimed.2009.10.027, PMID: 19914759

[21] Diamant AL Hays RD Morales LS Ford W Calmes D Asch S. Delays and unmet need for health care among adult primary care patients in a restructured urban public health system. Am J Public Health. (2004) 94:783–9. doi: 10.2105/AJPH.94.5.783, PMID: 15117701 PMC1448338

[22] Braveman P Gruskin S. Defining equity in health. J Epidemiol Community Health. (2003) 57:254–8. doi: 10.1136/jech.57.4.254, PMID: 12646539 PMC1732430

[23] Han S Nam S. A study on the categorization of unmet healthcare needs influencing factors for older adults with disabilities living in the community. Health Soc Welf Rev. (2021) 41:26–43. doi: 10.15709/hswr.2021.41.4.26

[24] Kim YM. Analysis of research trends for unmet medical care. HSS21. (2020) 11:997–1008. doi: 10.22143/HSS21.11.3.71

[25] Kim ES Eun SJ. Trend of unmet medical need and related factors using panel data. J Converg Inf Technol. (2020) 10:229–36. doi: 10.22156/CS4SMB.2020.10.09.229

[26] World Health Organization (WHO) (2010). A conceptual framework for action on the social determinants of health. Available at: https://apps.who.int/iris/handle/10665/44489.

[27] Ko MI Kim K. Analyses on the changes in the spatial distribution of Korean local extinction risk. J Korean Cartogr Assoc. (2021) 21:65–74. doi: 10.16879/jkca.2021.21.1.065

[28] Yoon B Kim K. A study on the spatial distribution and the actual status of the regional extinction risk in Chungcheongbuk-do. J Korean Urban Geogr Soc. (2022) 25:55–65. doi: 10.21189/JKUGS.25.3.5

[29] Yun J Cho Y. Analysis of changes in the risk of extinction in Haengjeong-ri unit villages using the local extinction index: a case study on Chungcheongnam-do. J Korean Soc Rural Plan. (2021) 27:103–16. doi: 10.7851/ksrp.2021.27.1.103

[30] Chang I Woo H Lim J Sohn H Park J (2020). Regional demographic changes and policy responses in Korea. Korea Institute for Health and Social Affairs. Available at; https://www.kihasa.re.kr/en/publish/paper/research/view?seq=36263.

[31] Pheakdey S Chan N Kolesar RJ Chak C. Improving health service quality in the Kingdom of Cambodia: a policy perspective. Asia Pac J Public Health. (2020) 32:426–9. doi: 10.1177/1010539520957841, PMID: 32929980 PMC7768884

[32] Shin HR Lim YG Han KM. The influence of medical expenditure on unmet needs for health care: focused on the moderating effect of private health insurance. Korean Assoc Reg Stud. (2014) 22:25–48. doi: 10.22921/jrs.2014.22.3.002

[33] Andersen R. A behavioral model of families’ use of health services. Chicago: Center for Health Administration Studies, University of Chicago (1968). 25 p.

[34] Chung SH. Population change and risk of regional extinction in Gangwon Province. J Soc Sci. (2019) 58:3–22. doi: 10.22418/JSS.2019.6.58.1.3

[35] Anselin L Bera AK. Spatial dependence in linear regression models with an introduction to spatial econometrics In: Ullah A Giles DEA, editors. Handbook of applied economic statistics. New York: Marcel Dekker (1998). 237–89.

[36] Moran PA. Notes on continuous stochastic phenomena. Biometrika. (1950) 37:17–23. doi: 10.1093/biomet/37.1-2.17, PMID: 15420245

[37] Tu J Xia ZG. Examining spatially varying relationships between land use and water quality using geographically weighted regression I: model design and evaluation. Sci Total Environ. (2008) 407:358–78. doi: 10.1016/j.scitotenv.2008.09.031, PMID: 18976797

[38] Raudenbush SW Bryk AS. Hierarchical linear models: Applications and data analysis methods 1. London: Sage (2002).

[39] Lee H-Y Noh S-C. Advanced Statistical Analysis. Gyeonggi: Beomunsa. (2012), 434–46.

[40] Glaser D Hastings RH. An introduction to multilevel modeling for anesthesiologists. Anesth Analg. (2011) 113:877–87. doi: 10.1213/ANE.0b013e3182198a01, PMID: 21680861

[41] Akaike H. A new look at the statistical model identification. IEEE Trans Autom Contr. (1974) 19:716–23. doi: 10.1109/TAC.1974.1100705

[42] Jo DG. GIS and geographically weighted regression in the survey research of small areas. Korean Assoc Survey Resources. (2009) 10:1–19.

[43] Lee KS Choi YJ. The effects of rival hospitals on the number of patients in a tertiary hospital. J Korean Oper Res Manag Sci Soc. (2012) 37:211–23. doi: 10.7737/JKORMS.2012.37.4.211

[44] Lim JH. Analysis of unmet medical need status based on the Korean health panel. Health Soc Sci. (2013) 34:237–56.

[45] Choi HY Ryu SY. Factors associated with the types of unmet health care needs among the elderly in Korea. Health Serv Manag. (2017) 11:65–79. doi: 10.12811/kshsm.2017.11.2.065

[46] Jang HY Lee H. Factors influencing unmet healthcare needs among elderly living alone. Korean Data Anal Soc. (2017) 19:3317–29. doi: 10.37727/jkdas.2017.19.6.3317

[47] Kwon YG Choi MK. Spatial analysis of the relationship between out-of-pocket expenditure and socioeconomic status in South Korea. Geospat Health. (2023) 18:1. doi: 10.4081/gh.2023.1175 37246540

[48] Bahn YK (2015). Study on factors related to the unmet healthcare needs in single-person households [dissertation]. Yonsei University Health System Repository. Available at: https://ir.ymlib.yonsei.ac.kr/handle/22282913/149032

[49] Gredler GR Collins R. The credential society: an historical sociology of education and stratification. New York: Academic Press (1979).

[50] Song HY Choi JW Park EC. The effect of economic participatory change on unmet needs of health care among Korean adults. Health Policy Manag. (2015) 25:11–21. doi: 10.4332/KJHPA.2015.25.1.11

[51] Hwang JN. Income-related inequality in unmet healthcare needs-implication of equity. Korean J Health Educ Promot. (2017) 34:83–97. doi: 10.14367/kjhep.2017.34.4.83

[52] Kim SA Seo Y Woo KS Shin Y. A systematic review of studies on current status and influencing factors of unmet medical needs in Korea. ACSW. (2019) 62:53–92. doi: 10.47042/ACSW.2019.02.62.53

[53] Song E Park E Choi SK. Unmet needs among patients with hypertension or diabetes during the COVID-19 pandemic by household income 42. Korea Institute for Health and Social Affairs. (2022) 3:246–59. doi: 10.15709/hswr.2022.42.3.246

[54] Idler EL Kasl SV. Self-ratings of health: do they also predict change in functional ability? J Gerontol B Psychol Sci Soc Sci. (1995) 50:S344–53. doi: 10.1093/geronb/50b.6.s344, PMID: 7583813

[55] Kim KS Ho L. Household catastrophic health expenditure and unmet needs depending on the types of health care system. Soc Welf. Policy. (2012) 39:255–79. doi: 10.15855/swp.2012.39.4.255

[56] Chang I. Does the spatial distribution of hospitals and clinics affect the unmet medical care of the elderly? (using evidence from Korea). J Korean Off Stat. (2020) 25:49–77. doi: 10.22886/jkos.2020.25.2.49

[57] Taqi M Bidhuri S Sarkar S Ahmad WS Wangchok P. Rural healthcare infrastructural disparities in India: a critical analysis of availability and accessibility. J Multidiscip Res Healthc. (2017) 3:125–49. doi: 10.15415/jmrh.2017.32011

[58] United Nations Development Programme (2017). UNDP support to the implementation of sustainable development goal 3: Ensure healthy lives and promote well-being for all at all ages. https://www.undp.org/sites/g/files/zskgke326/files/publications/SDG-3%20Health.pdf (Accessed June 14, 2024).

[59] Byun P Cha E Kim SH Lim SY Park SY Lim JY. Policy directions of central places for efficiently using public financial resources and securing people’s welfare. Korea Research Institute Hum. Settle. (2016) 16.

[60] Im S Byun P (2014). Trends and implications of underserved area policies in Japan – focusing on the establishment of small hubs and the formation of regional living clusters. National Land Brief Korea Research Institute for Human Settlements. https://www.dbpia.co.kr/pdf/pdfView.do?nodeId=NODE02506760&googleIPSandBox=false&mark=0&ipRange=false&accessgl=Y&language=ko_KR&hasTopBanner=true (Accessed December 8, 2014).

[61] Ju SH. Analysis of the priorities of local extinction response policy and policy implications. Korean J Local Gov Stud. (2023) 26:115–36. doi: 10.20484/klog.26.4.5

[62] Arbia G Petrarca F. Effects of MAUP on spatial econometric models. Lett Spat Resour Sci. (2011) 4:173–85. doi: 10.1007/s12076-011-0065-9

