# Supplementary material for: Unmet healthcare needs and the local extinction index: an analysis of regional disparities impacting South Korea’s older adults
Source: Front Public Health. 2024 Aug 1;12:1423108. doi: 10.3389/fpubh.2024.1423108 (PMC11325592; doi:10.3389/fpubh.2024.1423108)
Supplement: Supplementary file 1 [file Data_Sheet_1.docx]

**Supporting information**

**Appendix captions**

**Appendix A. Choropleth map of the Local Extinction Index**

| **Years** | **2018** | **2019** |
| --- | --- | --- |
| **Local Extinction**  **Index** | 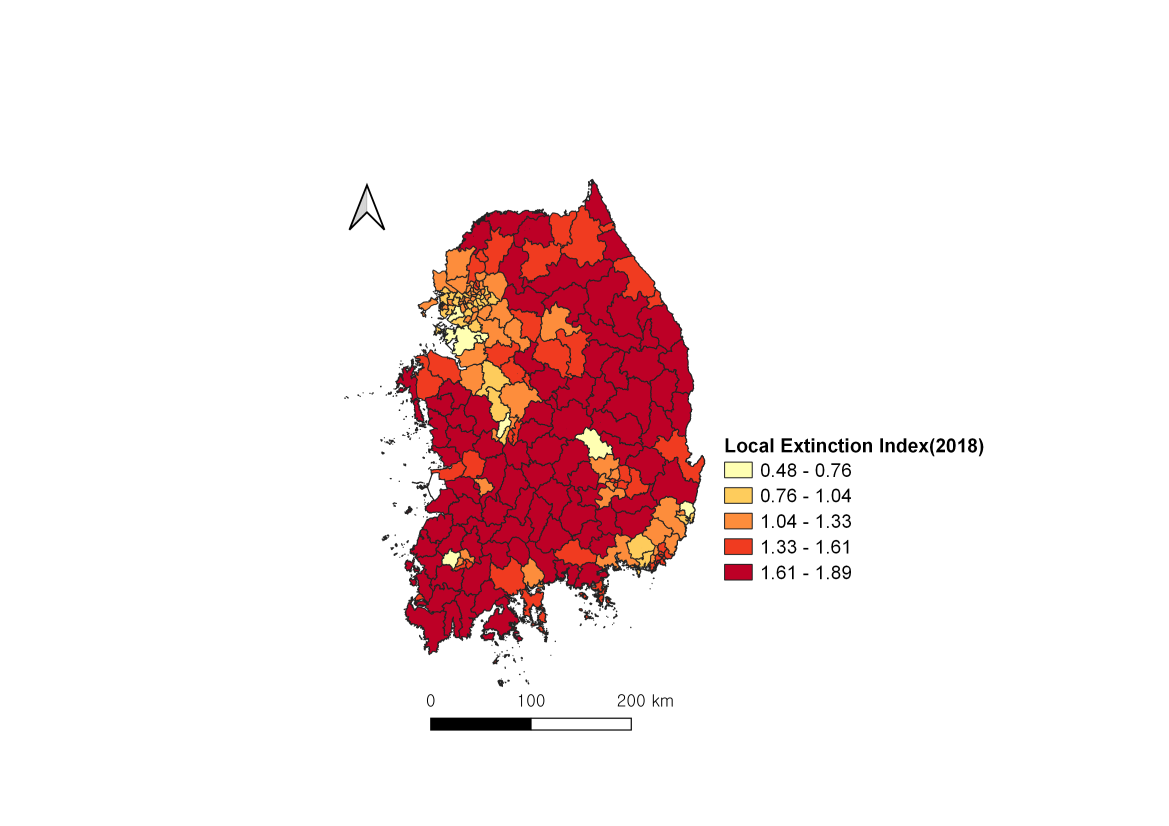 | 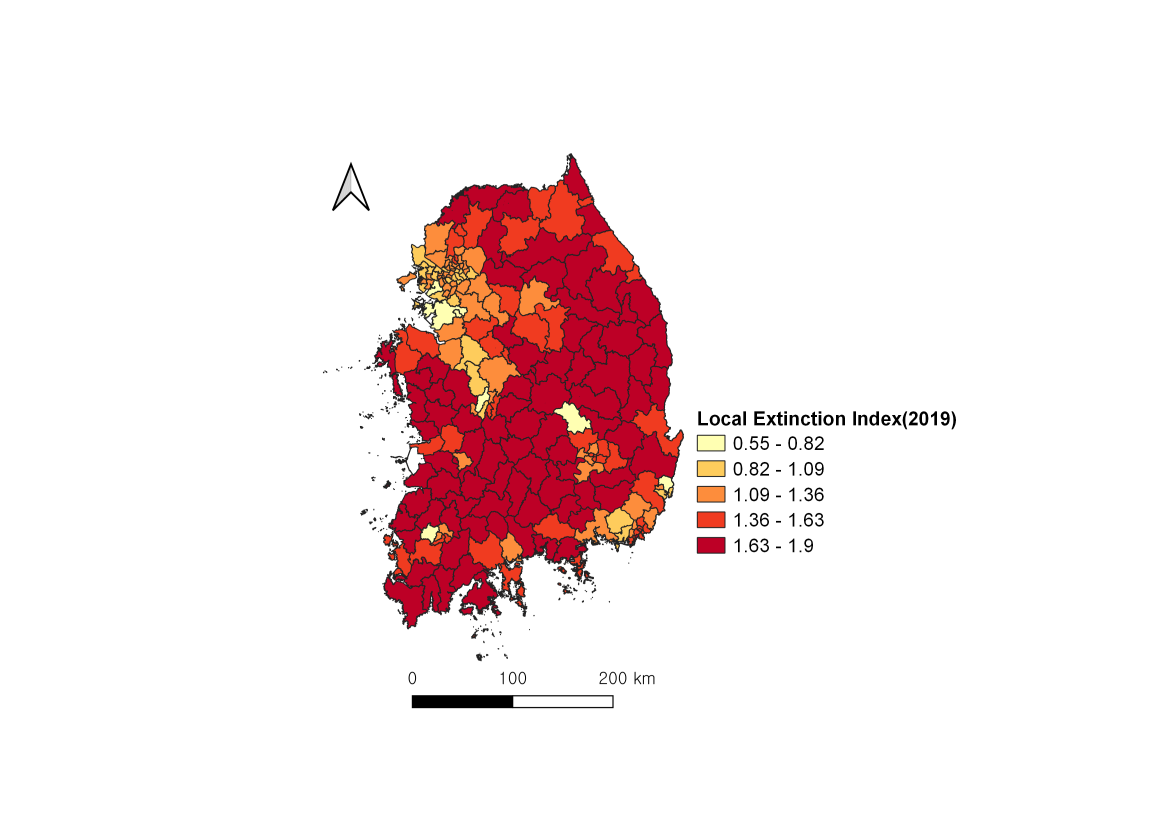 |

**Appendix B. Local Extinction Index by City from 2018 to 2019**

| 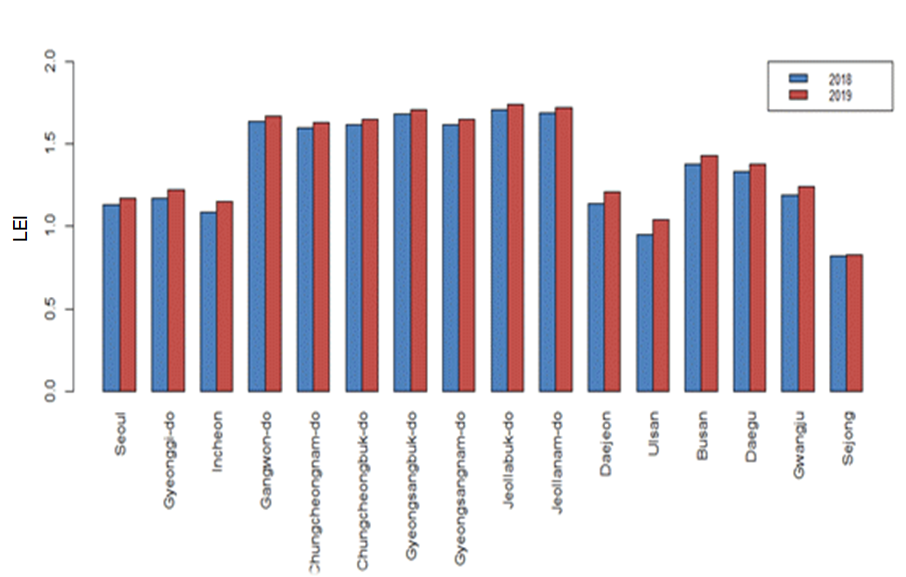 |
| --- |
